# Incorporating economies of scale in the cost estimation in economic evaluation of PCV and HPV vaccination programmes in the Philippines: a game changer?

**DOI:** 10.1186/s12962-018-0087-x

**Published:** 2018-02-20

**Authors:** Thanthima Suwanthawornkul, Naiyana Praditsitthikorn, Wantanee Kulpeng, Manuel Alexander Haasis, Anna Melissa Guerrero, Yot Teerawattananon

**Affiliations:** 1 Health Intervention and Technology Assessment Program, Ministry of Public Health, Nonthaburi, 11000 Thailand; 2 Department of Health Philippines, Pharmaceutical Division, National Center for Pharmaceutical Access and Management, 3/F Building 15, San Lazaro Compound, Rizal Avenue, Sta. Cruz, 1003 Manila, Philippines

**Keywords:** Cost estimation, Economic evaluation, Economies of scale, Human papillomavirus, Pneumococcal conjugate vaccine

## Abstract

**Background:**

Many economic evaluations ignore economies of scale in their cost estimation, which means that cost parameters are assumed to have a linear relationship with the level of production. Economies of scale is the situation when the average total cost of producing a product decreases with increasing volume caused by reducing the variable costs due to more efficient operation. This study investigates the significance of applying the economies of scale concept: the saving in costs gained by an increased level of production in economic evaluation of pneumococcal conjugate vaccines (PCV) and human papillomavirus (HPV) vaccinations.

**Methods:**

The fixed and variable costs of providing partial (20% coverage) and universal (100% coverage) vaccination programs in the Philippines were estimated using various methods, including costs of conducting questionnaire survey, focus-group discussion, and analysis of secondary data. Costing parameters were utilised as inputs for the two economic evaluation models for PCV and HPV. Incremental cost-effectiveness ratios (ICERs) and 5-year budget impacts with and without applying economies of scale to the costing parameters for partial and universal coverage were compared in order to determine the effect of these different costing approaches.

**Results:**

The program costs of the partial coverage for the two immunisation programs were not very different when applying and not applying the economies of scale concept. Nevertheless, the program costs for universal coverage were 0.26 and 0.32 times lower when applying economies of scale compared to not applying economies of scale for the pneumococcal and human papillomavirus vaccinations, respectively. ICERs varied by up to 98% for pneumococcal vaccinations, whereas the change in ICERs in the human papillomavirus vaccination depended on both the costs of cervical cancer screening and the vaccination program. This results in a significant difference in the 5-year budget impact, accounting for 30 and 40% of reduction in the 5-year budget impact for the pneumococcal and human papillomavirus vaccination programs.

**Conclusions:**

This study demonstrated the feasibility and importance of applying economies of scale in the cost estimation in economic evaluation, which would lead to different conclusions in terms of value for money regarding the interventions, particularly with population-wide interventions such as vaccination programs. The economies of scale approach to costing is recommended for the creation of methodological guidelines for conducting economic evaluations.

## Background

Health expenditure has risen for many years worldwide parallel with the demand for health care services [1]. As a result, when making decisions on the use of limited health resources, policy makers need to consider not only the clinical benefits but also economic information, including value for money and the budget impact of particular health interventions and technologies [2]. Even though cost is an important parameter for economic analysis, researchers often pay little attention to identifying accurate and reliable cost information compared to clinical parameters [3]. In common with the production and delivery of technologies in many industries, the unit cost of health technologies and interventions is likely to be affected by scale due to the efficiency gained by an increased level of production. This results in a non-linear function of the cost of production of health services or health technologies in relation to the size of production. Despite this, a few economic evaluation studies have incorporated economies of scale in their analysis [4]. The *WHO’s Choosing Interventions that are Cost*-*Effective (CHOICE)* project recommends the application of economies and diseconomies of scale when estimating the costs and impacts of various interventions with different coverage levels [5, 6].

Further, the *Reference Case* developed by the International Decision Support Initiative (iDSI) underlines the need to apply economies of scale in cost estimation, where appropriate [7]. Although incorporating economies of scale in an analysis is very reasonable, it is methodologically challenging, especially in low- and middle-income countries where health information infrastructures have not been well established. Thus, this study aims to investigate the feasibility and significance of applying the economies of scale concept to the economic evaluation and budget impact analysis of economic models for pneumococcal conjugate vaccines (PCV) and human papillomavirus (HPV) vaccine in the Philippines. The vaccine cases were selected for this study because of two reasons. First, it has been well recognised that the cost of the supply chain and vaccine procurement can be significantly affected by the number of vaccinations [8]. Second, the Government of the Philippines set their milestone to increase budget allocation every year for expansion the newly introduced vaccines. Their main priorities are infants, children, women, and elderly persons nationwide [9]. Strengthening the evidence on financial sustainability through the finding from this study can support decision making in the expanded program on immunization.

## Methods

### Model structures

Two economic evaluation models used for previous economic evaluations of PCV and HPV in the Philippines were deployed in this study. Details of the models are described elsewhere in open-access journals [10, 11]. Briefly, the two models compared both the costs and outcome of the PCV and HPV vaccination with 0–1 years for both boys and girls, and 11 years and above for girls, respectively. The models compared the vaccination programmes with the current practices, i.e. do nothing in the case of PCV and cervical cancer screening (visual inspection with acetic acid—VIA) in the case of HPV. The lifetime time horizons with the discount rate of 3.5% for both costs and outcome in terms of quality-adjusted life years (QALYs) were used consistently across the two models. Because this study focuses on applying the economies of scale concept to costing estimations, the epidemiological intervention effectiveness and utility information have been unchanged.

### Fixed costs

Using the provider’s perspective, the costs of the vaccination programmes were divided into two groups, i.e. fixed costs and variable costs. The fixed costs included cold chain-related infrastructure investment, which means that the costs of a functioning cold chain system were independent from the target population proportion to be covered by the vaccine programme. In other words, the higher the number of vaccinations provided was, the lower was the cold chain vaccination cost attached to each vaccine provided. The data on the cold chain investment in the Philippines were gathered from the Department of Health-Family Health Office, Ministry of Health (personal communication from programme manager of the Expanded Program on Immunization, the Philippines). Since the cold chain is used to support three different vaccination programmes, namely PCV, HPV, and inactivated polio vaccine, this joint cost was divided according to the number of vaccine dosages currently under the cold chain system. The PCV programme accounts for 55% of the total investment and the HPV programme accounts for 25%. The costs are presented in Table 1.

**Table 1:** Estimating cold chain investment cost per annum in relation to each vaccination programme

Type of vaccine	No. of doses	Proportion	Investment in cold chain (USD per year)
Pneumococcal conjugate vaccine	6,600,000	0.557	790,000
Human papillomavirus vaccine	3,044,100	0.257	364,000
Inactivated polio vaccine	2,200,000	0.186	263,000
Total	11,844,100	1.00	1,417,000

### Variable costs

The variable costs included vaccine acquisition costs, wastage costs, and logistic and administration costs. Originally, it was planned that the vaccine acquisition costs would be derived from a price survey among the vaccine companies. Despite requests directly from the Pharmaceutical Division of the Department of Health Philippines to vaccine companies, information about vaccine costs and administrative costs was not forthcoming. As such, the researchers used the current procurement prices for the scenario regarding the current vaccine coverage, i.e. 90, 88 and 86% for the first, second, and booster dose of PCV, respectively, the correspondence based on the 2013 coverage rates for the DPT-HepB-Hib vaccination for the first two doses and for the measles vaccination administered at the same time as the booster dose [9], and 10% for the HPV vaccination programmes were assumed to correspond to the achieved 2012 incorporate rate of pharmacy administration services regarding the drug price reference index of the DOH [12]. The researchers assumed the cheapest price for vaccine acquisition for 100% coverage of the HPV vaccine using the current GAVI’s procurement prices (USD 10.30 for PCV10, USD 10.40 for PCV13, and USD 4.50 for HPV) [13, 14] and varied the prices between the current coverage and 100% coverage using a linear assumption. The vaccine wastage costs, and logistic and administration costs, were assumed to be at 25% of the vaccine acquisition costs according to the observed rates in Thailand [15]. These cost parameters are showed in Tables 2 and 3.

**Table 2:** Cost of PCV vaccination (USD) for different percentages of vaccination coverage

% vaccine coverage	No. of vaccinated children	Average fixed cost	Average variable costs						Total cost of PCV vaccination per dose	
		Cost of cold chain per vaccination	PCV10			PCV13			PCV10	PCV13
			Vaccine cost	Logistic and administration cost	Wastage cost	Vaccine cost	Logistic and administration cost	Wastage cost		
10	220,000	1.20	14.74	2.95	0.74	16.54	3.31	0.83	19.63	21.87
20	440,000	0.60	14.25	2.85	0.71	15.86	3.17	0.79	18.41	20.42
30	660,000	0.40	13.76	2.75	0.69	15.18	3.04	0.76	17.60	19.37
40	880,000	0.30	13.26	2.65	0.66	14.49	2.90	0.72	16.88	18.42
50	1,100,000	0.24	12.77	2.55	0.64	13.81	2.76	0.69	16.20	17.50
60	1,320,000	0.20	12.28	2.46	0.61	13.13	2.63	0.66	15.54	16.61
70	1,540,000	0.17	11.78	2.36	0.59	12.45	2.49	0.62	14.90	15.73
80	1,760,000	0.15	11.29	2.26	0.56	11.76	2.35	0.59	14.26	14.86
90	1,980,000	0.13	10.79	2.16	0.54	11.08	2.22	0.55	13.63	13.99
100	2,200,000	0.12	10.30	2.06	0.52	10.40	2.08	0.52	12.99	13.12

Not taking into account economies of scale, the unit cost of PCV10 and PCV13 was USD 44.73 and USD 50.16

*PCV* pneumococcal conjugate vaccine

**Table 3:** Cost of HPV vaccination (USD) for different percentages of vaccination coverage

% vaccine coverage	No. of vaccinated girls	Average fixed cost	Average variable costs			Total cost of HPV vaccination per dose
		Cost of cold chain per vaccination	Vaccine cost	Logistic and administration cost	Wastage cost	
10	101,470	1.2	15.1	3.0	0.8	20
20	202,940	0.6	13.9	2.8	0.7	18
30	304,410	0.4	12.8	2.6	0.6	16
40	405,880	0.3	11.6	2.3	0.6	15
50	507,350	0.2	10.4	2.1	0.5	13
60	608,820	0.2	9.2	1.8	0.5	12
70	710,290	0.2	8.0	1.6	0.4	10
80	811,760	0.1	6.9	1.4	0.3	9
90	913,230	0.1	5.7	1.1	0.3	7
100	1,014,700	0.1	4.5	0.9	0.2	6

Not taking into account economies of scale, the unit cost per dose of HPV was USD 20

*HPV* human papillomavirus vaccine

### Incorporating economies of scale

For the cervical cancer modelling, the economies of scale were also applied to VIA screening and cryotherapy for the early stage of cervical cancer detected by the screening programme. The fixed costs included training and medical devices, e.g. cryotherapy units. The variable costs included labour costs and consumable materials such as acetic acid, CO_2_, etc. The data were collected from the MOH and are shown in Table 4.

**Table 4:** Cost of cervical cancer screening (USD)

% screening coverage	No. of eligible women per year	Unit cost of VIA screening	Unit cost of cryotherapy	Total cost of cervical cancer screening per woman
10	139,941	24	9	33
20	279,882	12	5	18
30	419,824	16	6	23
40	559,765	12	5	18
50	699,706	10	5	14
60	839,647	12	5	18
70	979,588	10	5	15
80	1,119,530	12	5	18
90	1,259,471	11	5	16
100	1,399,412	10	5	14

Not taking into account economies of scale, the unit cost of cervical cancer screening was USD 35.44

*VIA* visual inspection with acetic acid

The treatment costs for pneumococcal infection, including its complications and cervical cancer for human papillomavirus infection, were collected in the Philippines and in Thailand when the data in the Philippines were not available. The details of these costs were available in previous publications [10, 11]. Because the treatment costs depend on general access to the health facilities for each individual, the researchers did not apply economies of scale in the costing estimation for the treatments.

## Results

The results are presented in terms of an incremental cost-effectiveness ratio (ICER) for each vaccination programme with different coverage scenarios. Because there are many options for cervical cancer prevention and control, two comparators were represented in the analysis: (1) HPV vaccination plus cervical cancer screening compared with cervical cancer screening alone; and (2) HPV vaccination alone compared with cervical cancer screening alone. The first comparator is in line with the current policy option in the Philippines, whereas the second comparison was made to highlight the impact of economies of scale approach to economic evaluation when both policy choices were applicable for the approach. The economic analysis applied the ceiling threshold of Php 120,000 (USD 2835) in line with previous policy decisions for determining the value of health investment in the Philippines context. If the ICER was below the ceiling threshold, the intervention was considered to be cost-effective. If the ICER was lower than zero in this study, the intervention was considered to be a cost-saving option. In addition, the government budget implications for each policy option during the next 5 years are presented. All costs are presented in US dollars, USD, for the year 2012 (Php 0.024 = USD 1).

Table 5 shows a significant difference in the ICERs of PCV compared to the programme with no vaccination. Applying an economies of scale approach to estimating the cost of the vaccination programme accounted for a 62 and 71% reduction in ICERs for low vaccination coverage and up to 97 and 98% for high vaccination coverage in PCV10 and PCV13, respectively. It is noteworthy that the ICERs declined sharply with vaccination coverage equal to or above 80% as a result of herd protection. Nevertheless, the ICERs with an economies of scale approach did not change that policy conclusion—that PCV represents good value for money in the Philippine context.

**Table 5:** Incremental cost-effectiveness ratio of PCV vaccination compared to no vaccination

% coverage	PCV10 (USD/QALY)			PCV13 (USD/QALY)		
	Without taking into account EoS approach	With EoS approach	% reduction of ICER	Without taking into account EoS approach	With EoS approach	% reduction of ICER
10^a^	2655	1052	62	1997	760	71
20^a^	2655	975	65	1997	697	74
30^a^	2655	923	67	1997	650	75
40^a^	2655	877	70	1997	609	77
50^a^	2655	834	72	1997	569	79
60^a^	2655	792	73	1997	530	80
70^a^	2655	750	75	1997	491	81
80^b^	1439	134	97	1162	31	98
90^b^	1533	151	97	1232	38	98
100^b^	1614	159	97	1292	38	98

Herd protection was considered at a vaccination coverage rate of 80%

*PCV* pneumococcal conjugate vaccine, *EoS* economies of scale, *ICER* incremental cost-effectiveness ratio

^a^Low vaccination coverage

^b^High vaccination coverage

**Fig. 1:**
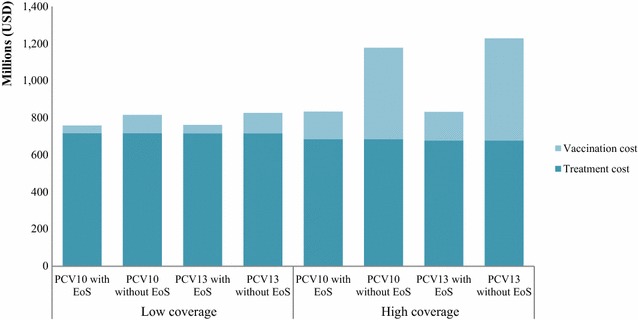
5-year budget impacts of PCV vaccination programmes with and without applying economies of scale. *PCV* pneumococcal conjugated vaccine, *EoS* economies of scale

Figure 1 illustrates the budget implications of the PCV vaccination programmes, and the treatment of pneumococcal-related infections using and not using the economies of scale approach. The figure indicates that the 5-year budget impacts of the vaccination programmes using the economies of scale approach accounted for only 30 and 40% of the budget estimation without using the economies of scale approach for high (100%) and low (20%) vaccination coverage, respectively.

Table 6 displays the ICERs of different coverage levels of the HPV vaccination programme on top of the cervical cancer screening compared to the different coverage of cervical cancer screening alone. The ICERs rely on coverage of cervical cancer screening—the lower the screening coverage was, the better was the value for the HPV vaccination programme given constant unit costs of vaccination and screening programmes (without taking into account economies of scale). These findings are contrary to the results represented in Table 7, in which the economies of scale approach was applied to the costing estimation of both policy options, i.e. vaccine plus cervical cancer screening and cervical cancer screening alone. Most of the scenarios, especially with high vaccination coverage, suggest that the vaccination programme plus cervical cancer screening is a cost-saving option. At low coverage, the vaccination plus cervical cancer screening policy remains a cost-effective option.

**Table 6:** Incremental cost-effectiveness ratio of HPV vaccination plus cervical cancer screening compared to cervical cancer screening alone: Incremental cost-effectiveness ratio of HPV vaccination and cervical cancer screening without taking into account economies of scale

ICER (USD/QALY)	Percent coverage of screening (not taking into account EoS approach)										
Percent coverage of HPV vaccine (not taking into account EoS approach)		10	20	30	40	50	60	70	80	90	100
	10	− 30^a^	120^b^	270^b^	440^b^	600^b^	770^b^	940^b^	1120^b^	1300^b^	1480^b^
	20	− 30^a^	120^b^	270^b^	440^b^	600^b^	770^b^	940^b^	1120^b^	1300^b^	1480^b^
	30	− 30^a^	120^b^	270^b^	440^b^	600^b^	770^b^	940^b^	1120^b^	1300^b^	1480^b^
	40	− 30^a^	120^b^	270^b^	440^b^	600^b^	770^b^	940^b^	1120^b^	1300^b^	1480^b^
	50	− 30^a^	120^b^	270^b^	440^b^	600^b^	770^b^	940^b^	1120^b^	1300^b^	1480^b^
	60	− 30^a^	120^b^	270^b^	440^b^	600^b^	770^b^	940^b^	1120^b^	1300^b^	1480^b^
	70	− 30^a^	120^b^	270^b^	440^b^	600^b^	770^b^	940^b^	1120^b^	1300^b^	1480^b^
	80	− 30^a^	120^b^	270^b^	440^b^	600^b^	770^b^	940^b^	1120^b^	1300^b^	1480^b^
	90	− 30^a^	120^b^	270^b^	440^b^	600^b^	770^b^	940^b^	1120^b^	1300^b^	1480^b^
	100	− 30^a^	120^b^	270^b^	440^b^	600^b^	770^b^	940^b^	1120^b^	1300^b^	1480^b^

*ICER* incremental cost-effectiveness ratio, *EoS* economies of scale, *HPV* human papillomavirus vaccine

^a^Cost-effective

^b^Cost-ineffective

**Table 7:** Incremental cost-effectiveness ratio of HPV vaccination plus cervical cancer screening compared to cervical cancer screening alone: Incremental cost-effectiveness ratio of HPV vaccination and cervical cancer screening with applying economies of scale

ICER (USD/QALY)	Percent coverage of screening (with EoS approach)										
Percent coverage of HPV vaccine (with EoS approach)		10	20	30	40	50	60	70	80	90	100
	10	− 30^a^	120^b^	280^b^	440^b^	600^b^	770^b^	950^b^	1120^b^	1300^b^	1480^b^
	20	− 470^a^	− 330^a^	− 190^a^	− 50^a^	100^b^	250^b^	400^b^	560^b^	720^b^	880^b^
	30	− 820^a^	− 690^a^	− 570^a^	− 440^a^	− 310^a^	− 170^a^	− 30^a^	110^b^	250^b^	400^b^
	40	− 1140^a^	− 1040^a^	− 920^a^	− 810^a^	− 690^a^	− 570^a^	− 450^a^	− 320^a^	− 190^a^	− 60^a^
	50	− 1460^a^	− 1370^a^	− 1270^a^	− 1170^a^	− 1060^a^	− 960^a^	− 850^a^	− 730^a^	− 620^a^	− 500^a^
	60	− 1780^a^	− 1700^a^	− 1610^a^	− 1520^a^	− 1430^a^	− 1340^a^	− 1240^a^	− 1140^a^	− 1050^a^	− 940^a^
	70	− 2090^a^	− 2020^a^	− 1950^a^	− 1870^a^	− 1800^a^	− 1720^a^	− 1640^a^	− 1550^a^	− 1470^a^	− 1380^a^
	80	− 2410^a^	− 2350^a^	− 2290^a^	− 2220^a^	− 2160^a^	− 2090^a^	− 2030^a^	− 1960^a^	− 1890^a^	− 1810^a^
	90	− 2720^a^	− 2670^a^	− 2620^a^	− 2570^a^	− 2520^a^	− 2470^a^	− 2420^a^	− 2360^a^	− 2300^a^	− 2250^a^
	100	− 3030^a^	− 2990^a^	− 2960^a^	− 2920^a^	− 2880^a^	− 2840^a^	− 2800^a^	− 2760^a^	− 2720^a^	− 2680^a^

*ICER* incremental cost-effectiveness ratio, *EoS* economies of scale, *HPV* human papillomavirus vaccine

^a^Cost-effective

^b^Cost-ineffective

Table 8 presents the ICERs for the vaccination programme plus cervical cancer screening using the economies of scale approach compared to the screening programme without taking into account the economies of scale approach. It suggests similar findings to Table 7.

**Table 8:** Incremental cost-effectiveness ratio of HPV vaccination plus cervical cancer screening compared to cervical cancer screening alone: Incremental cost-effectiveness ratio of HPV vaccination with applying economies of scale and cervical cancer screening without taking into account economies of scale

ICER (USD/QALY)	Percent coverage of screening (not taking into account EoS approach)										
Percent coverage of HPV vaccine (with EoS approach)		10	20	30	40	50	60	70	80	90	100
	10	− 30^a^	120^b^	270^b^	440^b^	600^b^	770^b^	940^b^	1120^b^	1300^b^	1480^b^
	20	− 470^a^	− 330^a^	− 190^a^	− 50^a^	100^b^	250^b^	400^b^	560^b^	720^b^	880^b^
	30	− 820^a^	− 690^a^	− 570^a^	− 440^a^	− 310^a^	− 170^a^	− 40^a^	110^b^	250^b^	400^b^
	40	− 1140^a^	− 1040^a^	− 920^a^	− 810^a^	− 690^a^	− 570^a^	− 450^a^	− 320^a^	− 190^a^	− 60^a^
	50	− 1460^a^	− 1370^a^	− 1270^a^	− 1170^a^	− 1060^a^	− 960^a^	− 850^a^	− 740^a^	− 620^a^	− 510^a^
	60	− 1780^a^	− 1700^a^	− 1610^a^	− 1520^a^	− 1430^a^	− 1340^a^	− 1240^a^	− 1150^a^	− 1050^a^	− 950^a^
	70	− 2090^a^	− 2020^a^	− 1950^a^	− 1870^a^	− 1800^a^	− 1720^a^	− 1640^a^	− 1550^a^	− 1470^a^	− 1380^a^
	80	− 2410^a^	− 2350^a^	− 2290^a^	− 2220^a^	− 2160^a^	− 2100^a^	− 2030^a^	− 1960^a^	− 1890^a^	− 1820^a^
	90	− 2720^a^	− 2670^a^	− 2620^a^	− 2570^a^	− 2520^a^	− 2470^a^	− 2420^a^	− 2360^a^	− 2310^a^	− 2250^a^
	100	− 3030^a^	− 2990^a^	− 2960^a^	− 2920^a^	− 2880^a^	− 2850^a^	− 2810^a^	− 2770^a^	− 2720^a^	− 2680^a^

*ICER* incremental cost-effectiveness ratio, *EoS* economies of scale, *HPV* human papillomavirus vaccine

^a^Cost-effective

^b^Cost-ineffective

Table 9 provides different findings—that without taking into account the economies of scale approach for the vaccination programme plus cervical cancer screening but with only the cervical cancer screening programme, the HPV vaccination plus cervical cancer screening policy was cost-ineffective in the Philippines except at 10% coverage for the screening programme. The higher the screening coverage was, the worse was the value for money of the vaccination programme and this indicated that the screening programme is a better choice for the Philippines.

**Table 9:** Incremental cost-effectiveness ratio of HPV vaccination plus cervical cancer screening compared to cervical cancer screening alone: Incremental cost-effectiveness ratio of HPV vaccination without taking into account economies of scale and cervical cancer screening with applying economies of scale

ICER (USD/QALY)	Percent coverage of screening (with EoS approach)										
Percent coverage of HPV vaccine (not taking into account EoS approach)		10	20	30	40	50	60	70	80	90	100
	10	− 30^a^	120^b^	280^b^	440^b^	600^b^	770^b^	950^b^	1120^b^	1300^b^	1480^b^
	20	− 30^a^	120^b^	280^b^	440^b^	600^b^	770^b^	950^b^	1120^b^	1300^b^	1480^b^
	30	− 30^a^	120^b^	280^b^	440^b^	600^b^	770^b^	950^b^	1120^b^	1300^b^	1480^b^
	40	− 30^a^	120^b^	280^b^	440^b^	600^b^	770^b^	950^b^	1120^b^	1300^b^	1480^b^
	50	− 30^a^	120^b^	280^b^	440^b^	600^b^	770^b^	950^b^	1120^b^	1300^b^	1480^b^
	60	− 30^a^	120^b^	280^b^	440^b^	600^b^	770^b^	950^b^	1120^b^	1300^b^	1480^b^
	70	− 30^a^	120^b^	280^b^	440^b^	600^b^	770^b^	950^b^	1120^b^	1300^b^	1480^b^
	80	− 30^a^	120^b^	280^b^	440^b^	600^b^	770^b^	950^b^	1120^b^	1300^b^	1480^b^
	90	− 30^a^	120^b^	280^b^	440^b^	600^b^	770^b^	950^b^	1120^b^	1300^b^	1480^b^
	100	− 30^a^	120^b^	280^b^	440^b^	600^b^	770^b^	950^b^	1120^b^	1300^b^	1480^b^

*ICER* incremental cost-effectiveness ratio, *EoS* economies of scale, *HPV* human papillomavirus vaccine

^a^Cost-effective

^b^Cost-ineffective

**Fig. 2:**
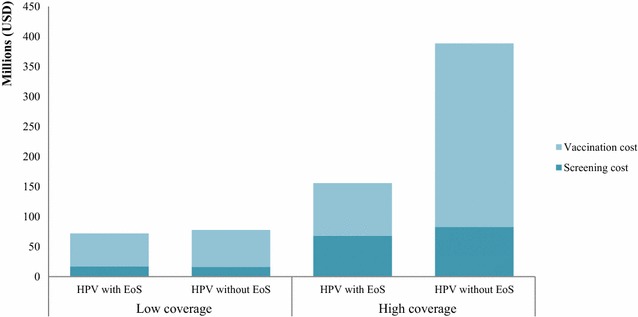
5-year budget impacts of HPV vaccination programmes with and without applying economies of scale. *HPV* human papillomavirus vaccine, *EoS* economies of scale

Figure 2 displays the budget impact of the HPV vaccination programme plus cervical cancer screening with and without taking into account the economies of scale approach. This indicates that in applying the economies of scale approach for economic evaluation, the 5-year budget impacts were 40 and 93% of the estimation without applying economies of scale at high (100%) and low (20%) vaccination plus screening coverage, respectively.

Tables 10, 11, 12, and 13 illustrate the impact of economies of scale in terms of ICERs when comparing the HPV vaccination programme alone with the cervical cancer screening alone. They show a higher impact of the economies of scale approach compared to Tables 6, 7, 8, and 9, resulting in preferable conclusion toward cervical cancer screening, particularly when high coverage of cervical cancer screening is compared to low coverage of HPV vaccination.

**Table 10:** Incremental cost-effectiveness ratio of HPV vaccination alone compared to cervical cancer screening alone: Incremental cost-effectiveness ratio of HPV vaccination and cervical cancer screening without taking into account economies of scale

ICER (USD/QALY)	Percent coverage of screening (not taking into account EoS approach)										
Percent coverage of HPV vaccine (not taking into account EoS approach)	% coverage	10	20	30	40	50	60	70	80	90	100
	10	1860^b^	− 11100^a^	− 3480^a^	− 2540^a^	− 2140^a^	− 1890^a^	− 1720^a^	− 1580^a^	− 1450^a^	− 1340^a^
	20	410^b^	1670^b^	6730^c^	− 15430^a^	− 5080^a^	− 3420^a^	− 2700^a^	− 2280^a^	− 1990^a^	− 1770^a^
	30	170^b^	670^b^	1510^b^	3230^c^	9470^c^	− 30430^a^	− 7260^a^	− 4470^a^	− 3330^a^	− 2690^a^
	40	60^b^	370^b^	780^b^	1350^b^	2250^b^	3940^c^	8580^c^	114020^c^	− 12250^a^	− 6170^a^
	50	10^b^	230^b^	490^b^	810^b^	1210^b^	1750^b^	2520^b^	3800^c^	6410^c^	15380^c^
	60	− 30^a^	140^b^	330^b^	550^b^	790^b^	1080^b^	1420^b^	1850^b^	2410^b^	3200^c^
	70	− 50^a^	90^b^	240^b^	400^b^	570^b^	750^b^	950^b^	1180^b^	1430^b^	1720^b^
	80	− 70^a^	50^b^	170^b^	300^b^	430^b^	560^b^	700^b^	840^b^	990^b^	1140^b^
	90	− 80^a^	20^b^	120^b^	230^b^	330^b^	430^b^	540^b^	640^b^	740^b^	830^b^
	100	− 90^a^	0^b^	90^b^	170^b^	260^b^	340^b^	430^b^	500^b^	570^b^	640^b^

*ICER* incremental cost-effectiveness ratio, *EoS* economies of scale, *HPV* human papillomavirus vaccine

^a^Cost-effective

^b^Cost-ineffective

^c^Highly cost-ineffective

**Table 11:** Incremental cost-effectiveness ratio of HPV vaccination alone compared to cervical cancer screening alone: Incremental cost-effectiveness ratio of HPV vaccination and cervical cancer screening with applying economies of scale

ICER (USD/QALY)	Percent coverage of screening (with EoS approach)										
Percent coverage of HPV vaccine (with EoS approach)	% coverage	10	20	30	40	50	60	70	80	90	100
	10	− 310^a^	− 11450^a^	− 2230^a^	− 2620^a^	− 2760^a^	− 1960^a^	− 2150^a^	− 1640^a^	− 1790^a^	− 1910^a^
	20	− 810^a^	760^b^	1530^b^	− 11420^a^	− 5400^a^	− 2810^a^	− 2870^a^	− 1960^a^	− 2130^a^	− 2230^a^
	30	− 1140^a^	− 520^a^	− 840^a^	590^b^	5880^c^	− 12850^a^	− 5170^a^	− 2330^a^	− 2510^a^	− 2600^a^
	40	− 1450^a^	− 1110^a^	− 1420^a^	− 940^a^	− 160^a^	− 750^a^	1480^b^	1350^b^	− 3400^a^	− 3150^a^
	50	− 1750^a^	− 1540^a^	− 1840^a^	− 1600^a^	− 1290^a^	− 1830^a^	− 1470^a^	− 2640^a^	− 2450^a^	− 1840^a^
	60	− 2050^a^	− 1920^a^	− 2190^a^	− 2070^a^	− 1910^a^	− 2370^a^	− 2250^a^	− 3010^a^	− 3020^a^	− 3050^a^
	70	− 2350^a^	− 2270^a^	− 2530^a^	− 2460^a^	− 2390^a^	− 2780^a^	− 2750^a^	− 3320^a^	− 3380^a^	− 3450^a^
	80	− 2650^a^	− 2610^a^	− 2850^a^	− 2820^a^	− 2800^a^	− 3150^a^	− 3160^a^	− 3630^a^	− 3700^a^	− 3790^a^
	90	− 2950^a^	− 2930^a^	− 3160^a^	− 3170^a^	− 3170^a^	− 3490^a^	− 3530^a^	− 3930^a^	− 4020^a^	− 4110^a^
	100	− 3250^a^	− 3250^a^	− 3470^a^	− 3500^a^	− 3520^a^	− 3820^a^	− 3870^a^	− 4230^a^	− 4320^a^	− 4420^a^

*ICER* incremental cost-effectiveness ratio, *EoS* economies of scale, *HPV* human papillomavirus vaccine

^a^Cost-effective

^b^Cost-ineffective

^c^Highly cost-ineffective

**Table 12:** Incremental cost-effectiveness ratio of HPV vaccination alone compared to cervical cancer screening alone: Incremental cost-effectiveness ratio of HPV vaccination with applying economies of scale and cervical cancer screening without taking into account economies of scale

ICER (USD/QALY)	Percent coverage of screening (not taking into account EoS approach)										
Percent coverage of HPV vaccine (with EoS approach)	% coverage	10	20	30	40	50	60	70	80	90	100
	10	1860^b^	− 11100^a^	− 3480^a^	− 2540^a^	− 2140^a^	− 1890^a^	− 1720^a^	− 1580^a^	− 1450^a^	− 1340^a^
	20	− 170^a^	700^b^	4160^c^	− 10880^a^	− 3830^a^	− 2680^a^	− 2170^a^	− 1860^a^	− 1640^a^	− 1470^a^
	30	− 770^a^	− 540^a^	− 200^a^	470^b^	2770^b^	− 11610^a^	− 3190^a^	− 2130^a^	− 1670^a^	− 1380^a^
	40	− 1190^a^	− 1120^a^	− 1060^a^	− 1000^a^	− 950^a^	− 920^a^	− 970^a^	− 4110^a^	− 160^a^	− 260^a^
	50	− 1550^a^	− 1550^a^	− 1580^a^	− 1630^a^	− 1740^a^	− 1910^a^	− 2230^a^	− 2840^a^	− 4220^a^	− 9370^a^
	60	− 1890^a^	− 1930^a^	− 2000^a^	− 2090^a^	− 2230^a^	− 2420^a^	− 2700^a^	− 3100^a^	− 3710^a^	− 4680^a^
	70	− 2210^a^	− 2280^a^	− 2370^a^	− 2480^a^	− 2630^a^	− 2820^a^	− 3070^a^	− 3390^a^	− 3810^a^	− 4370^a^
	80	− 2530^a^	− 2610^a^	− 2720^a^	− 2840^a^	− 2990^a^	− 3180^a^	− 3400^a^	− 3680^a^	− 4020^a^	− 4430^a^
	90	− 2840^a^	− 2940^a^	− 3050^a^	− 3180^a^	− 3330^a^	− 3520^a^	− 3730^a^	− 3970^a^	− 4260^a^	− 4600^a^
	100	− 3150^a^	− 3250^a^	− 3370^a^	− 3510^a^	− 3660^a^	− 3840^a^	− 4040^a^	− 4270^a^	− 4530^a^	− 4820^a^

*ICER* incremental cost-effectiveness ratio, *EoS* economies of scale, *HPV* human papillomavirus vaccine

^a^Cost-effective

^b^Cost-ineffective

^c^Highly cost-ineffective

**Table 13:** Incremental cost-effectiveness ratio of HPV vaccination alone compared to cervical cancer screening alone: Incremental cost-effectiveness ratio of HPV vaccination without taking into account economies of scale and cervical cancer screening with applying economies of scale

ICER (USD/QALY)	Percent coverage of screening (with EoS approach)										
Percent coverage of HPV vaccine (not taking into account EoS approach)	% coverage	10	20	30	40	50	60	70	80	90	100
	10	− 310^a^	− 11450^a^	− 2230^a^	− 2620^a^	− 2760^a^	− 1960^a^	− 2150^a^	− 1640^a^	− 1790^a^	− 1910^a^
	20	− 220^a^	1730^b^	4100^c^	− 15970^a^	− 6660^a^	− 3550^a^	− 3410^a^	− 2380^a^	− 2480^a^	− 2540^a^
	30	− 200^a^	700^b^	860^b^	3350^c^	12580^c^	− 31670^a^	− 9250^a^	− 4670^a^	− 4180^a^	− 3910^a^
	40	− 200^a^	390^b^	410^b^	1410^b^	3030^c^	4100^c^	11030^c^	119470^c^	− 15490^a^	− 9070^a^
	50	− 190^a^	240^b^	230^b^	840^b^	1660^b^	1820^b^	3280^c^	3990^c^	8180^c^	22910^c^
	60	− 190^a^	150^b^	140^b^	570^b^	1110^b^	1130^b^	1870^b^	1940^b^	3100^c^	4840^c^
	70	− 190^a^	100^b^	80^b^	420^b^	810^b^	790^b^	1270^b^	1240^b^	1860^b^	2630^b^
	80	− 190^a^	60^b^	40^b^	310^b^	620^b^	590^b^	940^b^	890^b^	1300^b^	1780^b^
	90	− 190^a^	30^b^	10^b^	240^b^	500^b^	460^b^	740^b^	680^b^	980^b^	1320^b^
	100	− 190^a^	0^b^	− 20^b^	190^b^	400^b^	370^b^	600^b^	530^b^	780^b^	1030^b^

*ICER* incremental cost-effectiveness ratio; *EoS* economies of scale; *HPV* human papillomavirus vaccine

^a^Cost-effective

^b^Cost-ineffective

^c^Highly cost-ineffective

## Discussion

The concept of economies of scale indicates that production and delivery unit costs diminish at greater scales of production [16, 17]. This study demonstrates the importance of using an economies of scale methodological approach in estimating the costs for the economic evaluations and budget impact analyses of the two vaccination programmes. This study assumes that economies of scale for vaccine unit costs yield different ICERs and budget impact estimations compared to conventional costing estimation in economic modelling, which assume constant average programme costs across different levels of service utilization. The new methodological approach may lead to different conclusions from the initial analysis undertaken and in this instance could contribute to alternative policy decisions regarding the adoption and roll-out of the PCV and HPV vaccines in the national vaccination programme in the Philippines. As a result, we believe that using economies of scale in costing estimation for economic evaluations and budget impact analyses is an appropriate approach and better categorises the nature of the problems regarding the decisions that policy makers face in the Philippines.

This is very important, especially in counties that are currently responsible for paying for the vaccine in their vaccination programmes or graduating from GAVI alliance. Further, it demonstrates the substantial impact on vaccine utilisation that GAVI-negotiated pricing could have in countries that do not receive direct GAVI support. Thus, it is in the interest of GAVI and other institutions at national and international levels concerned with improving access to vaccination to increase active support for advancing analytical methods that incorporate economies of scale in economic evaluation and budget impact analysis. These methodological advancements would also better inform National Immunization Technical Advisory Groups (NITAGs) and relevant public health authorities regarding the value for money and budget implications of the vaccine investment. Moreover, this approach is likely to be generalizable to the analysis of other types of technology and interventions beyond the vaccine programme area.

A key finding of this study is that incorporating economies of scale in the cost estimation in economic evaluation yielded higher magnitude of the value for health, especially with high vaccination coverage, in comparison without taking into account economies of scale. Our findings are in line with a systematic review of malaria control intervention conducted by White et al. [18]. The review indicated the effect of the scale of study on estimates of costs based on the number of beneficiaries or patients and concluded that economies of scale may result in cost savings per unit when an intervention is widely implemented. Our study adds to the very limited evidence about the relationship and impact of cost and scale of health interventions in terms of determining resource allocation, especially in resource-limited settings. We are aware that our results should be replicated to draw more concrete conclusions. Yet, resent research showed there is a higher tendency to find a positive result due to taking economies of scale than diseconomies and constant economies of scale [19]. However, results still vary across the wide range of settings and the selected outputs. Further studies may apply more accurate data in order to contribute to more productive output for the concept of incorporating economies of scale in cost estimation.

This study has some limitations, mainly related to assumptions required, due to incomplete information on how costs change in relation to volume. In particular, the relationship between the unit cost of vaccine at different levels of vaccine coverage has been approximated using a linear relationship where increasing coverage results in proportionate price reductions. Diseconomies of scale (where the unit cost actually increases with increasing volume) [20] have not been considered in this analysis. Although unit prices for vaccines are unlikely to be affected by diseconomies of scale, geographical and administrative issues may cause some diseconomies, particularly where near universal vaccine coverage is attempted. Second, this study only adopts the government perspective and ignores direct non-medical costs and indirect costs. However, many indirect costs, such as patient travel costs to access health facilities, would be borne on a per patient basis and would be unlikely to change with the number of patients reached by a national programme.

## Conclusions

This analysis has highlighted the need for more research into the production cost function of vaccination programmes and related health services in order to more accurately capture costs at scale, ultimately facilitating better-informed decisions about access to health technologies and interventions.

## Abbreviations

ICERs: incremental cost-effectiveness ratios
CHOICE: WHO’s Choosing Interventions that are Cost-Effective project
iDSI: International Decision Support Initiative
PCV: pneumococcal conjugate vaccine
HPV: human papillomavirus vaccine
VIA: visual inspection with acetic acid
QALYs: quality-adjusted life years
USD: US dollars
DPT-HepB-Hib: Diphtheria, Tetanus, Pertussis, Hepatitis B recombinant and Haemophilus influenza type B combined vaccine
DOH: Department of Health
GAVI: Global Alliance for Vaccines and Immunization
CO_2_: carbon dioxide
MOH: Ministry of Health
EoS: economies of scale
NITAGs: National Immunization Technical Advisory Groups
